# Correlation of TP53 Overexpression and Clinical Parameters with Five-Year Survival in Oral Squamous Cell Carcinoma Patients

**DOI:** 10.7759/cureus.1401

**Published:** 2017-06-27

**Authors:** Syed A Ali, Hamza A Khan, Omar Irfan, Adeel Samad, Yumna Mirza, Muhammad S Awan

**Affiliations:** 1 Surgery, The Aga Khan University; 2 Medical College, The Aga Khan University; 3 Medicine, The Aga Khan University

**Keywords:** oral squamous cell carcinoma (oscc), tp53, 5 year survival

## Abstract

Introduction

TP53 mutation and overexpression have been correlated with poor survival in many cancers including oral squamous cell carcinoma (OSCC). We aim to understand the role of TP53 overexpression in OSCC in our population and correlate it with five-year survival to test its viability as a prognostic marker for OSCC patients.

Materials and methods

Patients with biopsy proven OSCC at Aga Khan University Hospital from January 2000 to January 2008 were recruited. Immunohistochemistry was used to establish TP53 status and the results were published. Following up on these patients, five-year data were collected and correlated with TP53 status and other clinicopathologic parameters.

Results

Overexpression of TP53 was not significantly associated with five-year survival (hazard ratio [HR]: 1.543; 95% CI: 0.911-2.612; p = 0.107).

Conclusion

Although we had proven statistical relevance when correlated with overall survival in our previous study, we were unable to extend the same relevance to TP53 overexpression when it comes to five-year survival.

## Introduction

Oral squamous cell carcinoma (OSCC) is ranked as the eighth most common cancer, with global incidence being 299,050 and 12,720 in Pakistan in 2012 [1]. An alarming statistic is that Karachi, the largest city of Pakistan, bears the highest incidence of oral cavity cancer in the world [2]. Moreover, a study conducted in Karachi stated that OSCC among low-income families had increased by 200% from 1998 to 2002. According to the reports published by Pakistan Medical Research Council (PMRC), OSCC is the most common cancer among males and ranks second highest among females [3]. The worldwide mortality for 2012 was 145,350, stressing upon the need for effective management of the disease.

Emphasizing on the importance of this disease and its devastating consequences on our population, research in the developmental mechanisms of OSCC holds immense importance. The progress of OSCC at the molecular level is still unclear but latest developments present it to be the result of environmental factors causing mutations in oncogenes and tumor suppressor genes, particularly those involved in cell proliferation, differentiation, apoptosis and deoxyribonucleic acid (DNA) repair [4].

Several risk factors are known to aid in the pathogenesis of OSCC: smoking, alcohol consumption, chewing of betel quid and areca nut, and human papillomavirus infection [5]. Approximately 600 million people engage in betel quid chewing worldwide [5], making it the fourth most common addictive substance used, following tobacco, alcohol, and caffeine [6]. A study piloted in a squatter settlement of Karachi exposed that 40% of the population used betel nut and tobacco regularly [7].

Among all the mutations found in OSCC, the TP53 tumor suppressor gene is most frequently mutated [8-9]. The TP53 gene is located on the short arm of chromosome 17 and prevents cancer formation by maintaining genomic stability and regulating the cell cycle. Different kinds of stresses including oncogenic stimuli, ionizing radiation, UV radiation, hypoxia, cytokines and growth factors can activate the TP53 through different pathways [10]. As the “guardian of the genome”- p53 protein can initiate DNA repair proteins and stop the cell cycle at the G1/S regulation point when the DNA is damaged or activate apoptosis if the DNA is damaged beyond repair. When mutated, it allows cells to progress through DNA damage checkpoints and these cells are allowed to replicate along with their harmful mutations.

It has been established by the International Cancer Genome Consortium that the missense mutation is the most commonly occurring type (79%) among all TP53 mutations [11]. Mutations in TP53 make it more stable and hence easy to detect by immunohistochemistry [12]. However, any loss-of-function mutation in the TP53 does not directly transform the cells into neoplastic cells as loss of guardian function has no direct effect on proliferation. Instead, it permits the DNA to get damaged easily leading to mutations, acquisition of oncogenes, and neoplastic activity.

To date, even after intense research in management options, the five-year survival rate for OSCC has not increased significantly [13]. There have been very few studies on OSCC five-year survival worldwide and no studies to date in Pakistan, to the best of our knowledge. There have been a number of studies on one- and two-year survival but it is difficult to use such data to form conclusions in cancers like OSCC as these patients usually live for up to more than two years which stresses the need for five-year survival studies. Furthermore, the association of TP53 mutation and five-year survival of OSCC patients is still unclear. Correlation of TP53 mutation with gender, age, OSCC risk factors like alcohol and tobacco, site and grading are currently being established in different researches around the world in order to provide detailed information on OSCC patient prognosis at the time of presentation.

We have previously carried out and published a study discussing TP53 expression in terms of overall survival which concerned the same patient data set [14]. Five-year follow-up data for these patients was unavailable at the time of that study, as they had been freshly recruited, thus the aim of that study was to understand TP53 expression in terms of overall survival, as the duration of survival varied greatly among the patients. Subsequently, the aim of this study is to continue work on these patients and establish any and all such correlations between TP53 mutation and OSCC patient prognosis using a fixed five-year survival rate as it is the optimum method to validate prognostic use of any marker.

## Materials and methods

The study was a case series including 140 patients with primary OSCC who had been diagnosed and treated at Aga Khan University Hospital (AKUH), Karachi, Pakistan from January 2000 to January 2008. The study was approved by the Ethical Review Committee at AKUH and written informed consent was taken from all patients prior to their induction in the study. Inclusion criteria consisted of complete clinicopathologic data and presence of five-year follow-up data. Clinicopathologic information on each case including gender, age, TP53 overexpression, use of betel nut and/or other substances, smoking or alcohol consumption either alone or in different combinations, the site of OSCC, American Joint Committee on Cancer (AJCC) staging, tumor size, nodal involvement and metastasis was collected from our previously conducted study [14]. Additional five-year follow-up was obtained for all patients and they were classified as either alive or dead after 60 months. Immunohistochemistry was used in our previously reported study to assess TP53 overexpression using primary antibody TP53 (mouse monoclonal anti-TP53, clone DO-7, Dako Cytomation, diluted 1:50).

The results were recorded by the study investigators and data was analyzed using SPSS version 19 (IBM, NY, USA). Potential associations between demographic, histological, clinical parameters and five-year survival were assessed. Follow-up times for each patient were measured in months and mean survival was calculated from the date of diagnosis until death or for 60 months, whichever occurred sooner. Univariate Cox analysis was done to investigate the effect of clinicopathologic factors on mean five-year survival and a multivariate analysis was done for AJCC staging and TP53 positivity. A Kaplan-Meier curve was plotted to assess the event point distribution over a period of 60 months.

## Results

Patient characteristics

The total sample size was 140, with 69 patients expiring (event) and 71 alive (censored) at the end of 60 months. In our study, 82 patients (59%) were males and 58 (41%) were females with a male-female ratio of 1.4:1 and 112 (80%) patients were >40 years of age. The most frequently occurring primary lesion location was cheek (86) followed by tongue (54), mandible (36), palate (14), retromandible (17) and the floor of the mouth (14). Additionally, the highest number of patients presented with AJCC stage II disease (34%), followed by stage III (27%), stage IV (22%) and stage I (19%). Based on the disease stage, 82 (59%) patients were treated with surgery and radiation both while 58 (41%) were treated with surgery alone. Most commonly used addictive substance for our patients was betel quid, followed by tobacco. Table 1 shows the demographic details of the patients.

**Table 1:** Patient demographics at five-year follow-up (n = 140).

		Event (n = 69)		Censored (n = 71)		Total (n = 140)	
		Count	Column N %	Count	Column N %	Count	Column N %
Gender	Male	40	58.0%	42	59.2%	82	58.6%
	Female	29	42.0%	29	40.8%	58	41.4%
Age group	<40 years	11	15.9%	17	23.9%	28	20.0%
	>= 40 years	58	84.1%	54	76.1%	112	80.0%
TP53 value	Positive	49	71.0%	45	63.4%	94	67.1%
	Negative	20	29.0%	26	36.6%	46	32.9%
Positivity	Mild	8	11.6%	11	15.5%	19	13.6%
	Moderate	15	21.7%	7	9.9%	22	15.7%
	Strong	26	37.7%	27	38.0%	53	37.9%
	Negative	20	29.0%	26	36.6%	46	32.9%
Histological classification of tumor	W-D-SCC	26	37.7%	29	40.8%	55	39.3%
	M-D-SCC	41	59.4%	38	53.5%	79	56.4%
	P-D-SCC	2	2.9%	4	4.1%	6	4.3%
AJCC_STAGE	I	9	13.0%	18	25.4%	27	19.3%
	II	18	26.1%	26	36.6%	44	31.4%
	III	22	31.9%	16	22.5%	38	27.1%
	IV	20	29.0%	11	15.5%	31	22.1%
Smoking	Yes	26	37.7%	25	41.7%	51	36.4%
	No	43	62.3%	35	58.3%	89	63.6%
Betel quid chewer	Yes	43	62.3%	39	54.9%	82	58.6%
	No	26	37.7%	32	45.1%	48	34.4%
Tobacco	Yes	20	29.0%	23	32.4%	43	30.7%
	No	49	71.0%	48	67.6%	97	69.3%
Cheek/tongue	Cheek	48	69.6%	38	53.5%	86	61.4%
	Tongue	21	30.4%	33	46.5%	54	38.6%
Palate	Yes	6	8.7%	8	11.3%	14	10.0%
	No	63	91.3%	63	88.7%	126	90.0%
Mandible	Yes	18	26.1%	18	25.4%	36	25.7%
	No	51	73.9%	53	74.6%	104	74.3%
Retromandibular	Yes	11	15.9%	6	8.5%	17	12.1%
	No	48	69.6%	51	71.8%	99	70.7%
	NA	10	14.5%	14	19.7%	24	17.1%
Floor of mouth	Yes	4	5.8%	10	14.1%	14	10.0%
	No	65	94.2%	61	85.9%	126	90.0%
Tonsil	Yes	1	1.4%	2	2.8%	3	2.1%
	No	68	98.6%	69	97.2%	137	97.9%
Nodal status physical	Palpable node	31	44.9%	26	36.6%	57	40.7%
	No palpable node	38	55.1%	45	63.4%	83	59.3%
Single/multiple nodes	Single	20	29.0%	19	26.8%	39	27.9%
	Multiple	11	15.9%	7	9.9%	18	12.9%
	NA	38	55.1%	45	63.3%	83	59.3%
Final T stage	T1	12	17.4%	21	29.6%	33	23.6%
	T2	31	44.9%	33	46.5%	64	45.7%
	T3	12	17.4%	10	14.1%	22	15.7%
	T4	14	20.3%	7	9.8%	21	15.0%
Final N stage	N0	43	62.3%	59	83.1%	102	72.9%
	N1	18	26.1%	8	11.3%	26	18.6%
	N2a	2	2.9%	1	1.4%	3	2.1%
	N2b	6	8.7%	3	4.2%	9	6.4%
Final M stage	M0	69	100.0%	71	100.0%	140	100.0%
Neck pathology	Negative	27	39.1%	44	62.0%	71	50.7%
	Positive	25	36.2%	13	18.3%	38	27.1%
	ND	17	24.6%	14	19.7%	31	22.1%

Out of 140 tumors, 94 (67%) patients were TP53 positive and 46 (33%) patients were TP53 negative. TP53 overexpression was not significantly correlated with any clinicopathologic parameters tested, such as AJCC staging, habits, histology and final T and N stages. A total of 54 patients presented with tongue as the primary site of tumor, with 51 patients having tumor in the anterior tongue, five in the posterior and two patients having both. However, a significant association was observed between the anterior tongue as the site of tumor and TP53 positivity (p = 0.038). Table 2 shows TP53 expression and clinicopathologic parameters.

**Table 2:** Correlation of P53 overexpression and clinicopathologic parameters.

Characteristic		P53 overexpression positive	P53 overexpression negative	Pearson Chi-Square value	p-value
AJCC staging	Stage I	21	6	5.247	0.155
	Stage II	33	11		
	Stage III	22	16		
	Stage IV	18	13		
Habits	Smoking/tobacco	35	16	0.090	0.956
	Pan/supari	57	25	0.612	0.736
	Naswar/chalia/gutka	28	15	0.242	0.886
Final T stage	Stage I	23	10	4.439	0.218
	Stage II	47	17		
	Stage III	11	11		
	Stage IV	13	8		
Final N stage	N0	70	32	0.560	0.756
	N1	17	9		
	N2	7	5		
Histology	W-D-SCC	38	17	0.894	0.640
	M-D-SCC	53	26		
	P-D-SCC	3	3		
Site of tumor	Anterior tongue	30	23	4.294	0.038
	Posterior tongue	4	1	0.389	0.533
	Palate	10	4	0.130	0.719
	Mandible	23	13	0.233	0.630
	Retromandibular	11	6	0.387	0.824
	Floor of mouth	9	5	0.058	0.810
	Tonsil	3	0	1.500	0.221

Furthermore, AJCC staging, final N stage and neck pathology were found statistically significant on univariate analysis (p = 0.003, p < 0.001 and p = 0.003, respectively) (Table 3). Moreover, AJCC staging remained significant (p = 0.003) on multivariate analysis using COX regression, while TP53 persisted statistically insignificant.

Mean and median survival time

AJCC stage I was associated with significantly longer survival (49.9 months, 95% CI: 41.9-57.9) as compared to stage IV (31.8 months, 95% CI: 22.9-40.6) as shown in Table 3. Additionally, the absence of neck pathology displayed significantly longer survival (50.2 months, 95% CI: 45.8-54.5). Table 3 shows the univariate and multivariate analysis.

**Table 3:** Univariate and multivariate analysis.

Univariate Analysis					
Factor		Hazard ratio or Exp(B)	95.0% CI for Exp(B)		Sig.
			Lower	Upper	
TP53 value	Positive	1			.265
	Negative	1.344	.798	2.262	
AJCC_STAGE	I	.326	.148	.716	.003
	II	.361	.191	.684	
	III	.560	.305	1.028	
	IV	1			
Final N stage	N0	.249	.105	.592	<0.001
	N1	.560	.221	1.417	
	N2a	2.573	.494	13.385	
Neck pathology	Negative	.584	.318	1.072	.003
	Positive	1.482	.799	2.747	
Multivariate Analysis					
		Adjusted hazard ratio Exp(B)	95.0% CI for Exp(B)		Sig.
			Lower	Upper	
AJCC_STAGE					.003
	I	1.114	0.500	2.481	
	II	1.823	0.837	3.972	
	III	3.309	1.497	7.316	
TP53 value	Positive	1.543	0.911	2.612	.107
	Negative	1			

TP53 positivity and survival

All clinical features in Table 1 were examined to determine any correlation with TP53 overexpression using Pearson Chi-square test and it was found that TP53 overexpression was not significantly correlated with any clinicopathologic factor (HR: 1.543; 95% CI: 0.911-2.612, p = 0.107). Additionally, TP53 overexpression was also not statistically significant on univariate analysis (p = 0.265) (Table 3). Furthermore, there was no marked difference in mean survival time of TP53 positive (44.258, 95% CI: 39.918-48.597) and TP53 negative (46.676, 95% CI: 40.314-53.039) patients. Table 4 shows mean and median survival time.

**Table 4:** Mean and median survival time.

	Mean^a^				Median			
	Estimate	Std. error	95% confidence interval		Estimate	Std. error	95% Confidence interval	
			Lower bound	Upper bound			Lower bound	Upper bound
Overall	45.079	1.821	41.511	48.648	60.000	.964	58.111	61.889
TP53 value								
Positive	44.258	2.214	39.918	48.597	60.000	2.669	54.769	65.231
Negative	46.676	3.246	40.314	53.039				
Positivity								
Mild	53.500	3.618	46.409	60.591	60.000			
Moderate	36.994	4.513	28.148	45.841	32.000	3.737	24.676	39.324
Strong	43.894	3.107	37.805	49.983	60.000	4.226	51.717	68.283
Negative	46.676	3.246	40.314	53.039				
AJCC_STAGE								
I	49.944	4.093	41.921	57.967				
II	51.417	2.432	46.652	56.183				
II	44.831	3.397	38.173	51.488	60.000	7.122	46.041	73.959
IV	31.809	4.514	22.962	40.656	19.000	9.947	0.000	38.496
Final T stage								
T1	50.711	3.441	43.966	57.456				
T2	46.970	2.474	42.122	51.819	60.000	1.360	57.335	62.665
T3	43.905	4.815	34.468	53.342	60.000	11.148	38.149	81.851
T4	32.813	5.390	22.249	43.376	26.000	11.006	4.429	47.571
Final N stage								
N0	49.019	1.938	45.220	52.818				
N1	37.211	4.386	28.614	45.808	32.000	4.718	22.753	41.247
N2a	12.500	.500	11.520	13.480	12.000			
N2b	28.857	8.385	12.423	45.292	19.000	6.547	6.169	31.831
Neck pathology								
Negative	50.211	2.208	45.884	54.539				
Positive	37.573	3.880	29.969	45.178	40.000	7.767	24.776	55.224
ND	42.464	4.081	34.465	50.462	60.000	11.262	37.927	82.073

**Figure 1:**
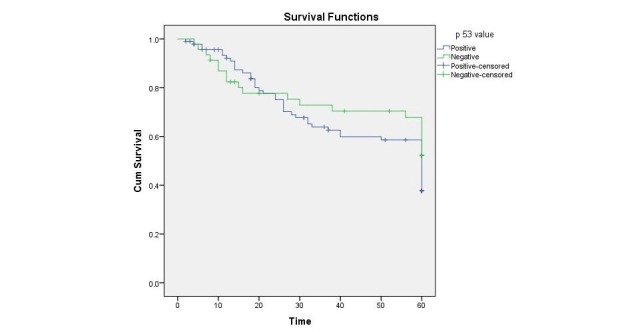
Kaplan-Meier curve depicting five-year survival of p53 positive and negative oral squamous cell carcinoma (OSCC) patients.

However, mild degree of positivity was found to be associated with longer survival (53.5, CI: 46.4-60.6) as compared to moderate degree of positivity (36.99, CI: 28.1-45.8). A Kaplan-Meier curve plotted as shown in Figure 1 for fixed five-year survival illustrated no significant difference in survival between TP53 positive and negative groups with Log Rank 0.239.

## Discussion

The carcinogenesis of the gastrointestinal tract epithelium is an intricate process that involves abnormalities of oncogenes and tumor suppressor genes [15-16]. Even today it is not clear why patients having identical tumor grading and staging behave differently when confronted with similar treatments. This may be due to unique molecular changes resulting from differing expression of certain genes.

Western countries have a high incidence of TP53 mutations in OSCC associated with these risk factors [17] with India [18] reported to have low incidence and Taiwan [19] with high incidence from Asia. We report no association of these risk factors including smoking, tobacco and betel quid usage with TP53 over expression.

Environmental insults are known to be one of the root causes of oral cancer, considering that this malignancy occurs almost exclusively in patients with tobacco, alcohol and/or betel quid usage and these agents are believed to cause a synergistic carcinogenic effect on the oral cavity [5]. The independent effect of these risk factors is difficult to ascertain considering that most subjects are using a combination of these risk factors and as a result multiple conflicting outcomes have been reported.

Of all related genes, TP53 has received foremost attention: Its prevalence in head and neck squamous cell cancer varies from 30% to 70% [20]. Almost all types of cancers are reported to have TP53 mutations, from 10% in hematopoietic malignancies to close to 100% in high-grade serous carcinoma of the ovary [21]. According to “The TP53 website” lung cancers have the highest TP53 mutation rate (70%) followed by colorectal (60%) and head and neck cancers (60%). Laryngeal squamous cell carcinoma is severely implicated in TP53 overexpression and so is the progression of non-malignant lesions to malignant ones, but for the prognostic significance of TP53 and observing its value for the entire head and neck squamous cell cancer group, results differ among studies.

In the past, there have been studies showing no correlation between TP53 expression and patient prognosis, regardless of whether the analysis involved only the invasive fronts of tumors [22], or tumors as a whole [23]. On the other hand in a study conducted on 16 patients with recurrent squamous cell carcinoma of the head and neck, a poor prognosis with positive TP53 expression was observed [24].

The presence of TP53 mutation was associated with a significantly decreased overall survival and even stronger association with disruptive mutations as shown by Poeta, et al. [25]. However, other multiple attempts have turned out inconclusive in correlating TP53 status and survival. The diversity of results may partly be due to: a diverse and small sample size, the tissue type analyzed and the methods used for analysis including antibodies, tissue treatment and the threshold set for positivity and negativity of stained sections [26].

In our study we observed that neither the histological differentiation nor the consumption of addictive substances or any other risk factors were associated with TP53 overexpression, only the location of tumor was significant for the anterior tongue showing most TP53 expression. AJCC staging, nodal status, and neck pathology were significantly associated with TP53 overexpression in univariate analysis but lost their significance in the multivariate analysis. This is in accordance with other reports including Field, et al. which reported no correlation between TP53 overexpression and any other clinicopathological parameters [27]. However, some previous reports [17] have described accumulating TP53 levels correlating with increasing histological severity and with anatomical site of the tumor.

## Conclusions

An ideal marker predicting tumor prognosis should indicate tumor formation when present and should exclude such possibility on absence and to identify such a marker the five-year survival analysis is indispensable. Our previous study highlighted the importance of TP53 mutation when dealt with overall survival ranging from months to years. Following up on these patients, however, displayed that TP53 overexpression could not be held statistically significant over five-year survival. This could be due to the differing anatomical location of tumor in our patient set, the type of exposure and sample size. However, it is unlikely that a single tumor marker can predict tumor progression since the development and progression of a tumor is a cumulative response to various factors. Therefore, it is equally important to look at the status of other factors closely related to TP53. Nevertheless, the TP53 pathway is a very important one in head and neck squamous cell cancer pathogenesis and also potentially in its management. We believe that TP53 overexpression estimation may eventually prove useful as a routine component of tumor assessment along with tumor staging.
